# A multi-label sentinel-2 dataset for deep learning-based energy, transport, and storage infrastructure mapping in Germany

**DOI:** 10.1016/j.dib.2026.113226

**Published:** 2026-09-03

**Authors:** Lukas Pasold, Luca Eisentraut, Felix Waigner, Ricardo Buettner

**Affiliations:** Chair of Hybrid Intelligence, Helmut-Schmidt-University/University of the Federal Armed Forces Hamburg, Holstenhofweg 85, 22043 Hamburg, Germany

**Keywords:** Classification, Remote sensing, Openstreetmap, Computer vision, Satellite Imagery

## Abstract

This article presents a multi-label Sentinel-2 image dataset addressing the limited availability of remote sensing benchmarks that reflect the real-world co-occurrence of energy, transport, and storage infrastructure. The collection contains 10,000 true-color RGB PNG image chips from across Germany. Each image is independently annotated for electrical substations, power plants, storage tanks, railway infrastructure, and major road infrastructure, allowing none, one, or multiple infrastructure types to occur within the same scene. Candidate locations and labels were derived from OpenStreetMap geometries. Samples were selected from retained linear and polygonal features, spatially balanced across Germany, and accepted only when their complete image footprints satisfied the defined intersection and non-overlap criteria. Contextually similar no-label samples were generated near infrastructure locations to provide challenging negative examples. Predefined spatial folds keep neighboring samples, associated no-label samples, and samples from the same local OpenStreetMap feature together, supporting reproducible evaluation while avoiding spatial leakage. The image chips were generated from Copernicus Sentinel-2 Level-2A bands B04, B03, and B02 using a maximum cloud-cover threshold of 10 percent and least-cloud-cover mosaicking. Each 224 × 224 pixel image is a resampled representation of a 512 m × 512 m footprint. The released files include the image collection and CSV metadata containing filenames, binary labels, and spatial fold assignments. Spatial five-fold baseline experiments demonstrate the dataset’s suitability for supervised multi-label classification, reproducible model comparison, and infrastructure mapping.

Specifications Table

**Table:** 

Subject	Computer Sciences
Specific subject area	Multi-label classification of energy, transport, and storage infrastructure in Sentinel-2 true-color imagery over Germany.
Type of data	Image (RGB PNG image chips), Table (CSV metadata tables containing binary labels and predefined spatial fold assignments).
Data collection	Sentinel-2 Level-2A imagery was used to create a multi-label infrastructure dataset for Germany, retrieved through the Copernicus Data Space Ecosystem Sentinel Hub Process API. Labels for substations, power plants, storage tanks, railways, and major roads were derived from OpenStreetMap. Samples were selected using spatial balancing, non-overlap filtering, and footprint-based exclusion rules. The 224 × 224 pixel images represent 512 m × 512 m footprints at a native spatial resolution of 10 m. Predefined spatial folds keep nearby and related samples together.
Data source location	Germany. Satellite imagery originates from the Copernicus Sentinel-2 Level-2A collection, providing observations from the Sentinel-2 satellite constellation. Infrastructure and contextual land-cover geometries originate from OpenStreetMap. Geographic coordinates were used internally for sampling and fold generation.
Data accessibility	Repository name: A Multi-Label Sentinel-2 Dataset for Deep Learning-Based Energy, Transport, and Storage Infrastructure Mapping in Germany Data identification number: 10.5281/zenodo.21263006 Direct URL to data: https://doi.org/10.5281/zenodo.21263006
Related research article	none

## 1 Value of the Data


 

• This collection supports reproducible benchmarking of remote sensing computer vision methods through 10,000 Sentinel-2 RGB image chips with multi-label annotations for energy, transport, and storage infrastructure. All chips share a fixed geographic footprint, image size, and acquisition protocol. Predefined spatial folds keep geographically related samples together, enabling controlled model comparisons while reducing spatial leakage between training and evaluation data.
• Spatially balanced sampling across Germany and non-overlap filtering reduce the overrepresentation of dense infrastructure clusters. The collection includes scenes from agricultural and open vegetation areas, urban environments, forests, industrial areas, and water or wetland contexts, supporting the evaluation of model robustness across heterogeneous environmental conditions.
• Realistic and challenging evaluation is supported by no-label samples generated near retained infrastructure locations rather than in randomly selected distant regions. These samples were accepted only when their complete footprints did not intersect retained label geometries or previously accepted sample footprints, reducing the likelihood that models rely primarily on broad regional or land-cover differences.
• The dataset provides a reusable benchmark for multi-label infrastructure mapping, comparisons of conventional computer vision models and geospatial foundation models, representation learning, hard-negative mining, transfer learning, domain adaptation, and uncertainty analysis. It may also support research on scalable infrastructure inventorying, large-area screening, and data-driven infrastructure monitoring.
• The multi-label design reflects the real-world co-occurrence of infrastructure systems within the same local landscape. By allowing each image chip to contain none, one, or multiple infrastructure labels, the dataset supports research on scene-level infrastructure recognition, label co-occurrence, and model behavior in visually complex mixed-infrastructure settings.

## 2 Background

Satellite imagery and machine learning enable spatial information on built environments and infrastructure to be derived at scales difficult to address through manual interpretation [1]. They support infrastructure inventorying, large-area screening, temporal comparison, and transferable geospatial models [2,3]. Energy, transport, and storage infrastructure are relevant applications because they form interdependent systems across urban, industrial, agricultural, and peri‑urban settings.

Recent publications show the value of task-specific Sentinel datasets for reusable machine-learning research, including road detection, multimodal image collections, and cloud and cloud-shadow benchmarks [[4], [5], [6]]. However, existing infrastructure-oriented Sentinel-2 datasets commonly focus on individual infrastructure categories or mutually exclusive labels. This does not fully reflect real-world infrastructure scenes, where several relevant infrastructure types may occur within the same local image footprint.

Mapping such infrastructure in Sentinel-2 imagery remains challenging because objects differ strongly in size, geometry, and appearance, while the visible Sentinel-2 bands used for RGB rendering have a native spatial resolution of 10 m [7]. Mixed industrial or peri‑urban scenes may include transport features, polygonal facilities, storage tanks, substations, power plants, vegetation, shadows, roads, railways, and buildings. This dataset therefore provides a reusable benchmark for multi-label infrastructure classification, supporting model comparisons, representation learning, hard-negative evaluation, and analyses across heterogeneous settings.

## 3 Data Description

The dataset [8] consists of Sentinel-2 image chips and accompanying comma-separated values (CSV) metadata tables. As summarized in Table 1, the collection contains 10,000 true-color image chips stored as RGB Portable Network Graphics (PNG) files. Each image has dimensions of 224 × 224 pixels and corresponds to a 512 m × 512 m geographic footprint in Germany. The images were rendered from Sentinel-2 Level-2A bands B04, B03, and B02, corresponding to the red, green, and blue channels, respectively [9].

**Table 1:** Components of the dataset.

Component	Format	Content
Image collection	RGB PNG	10,000 Sentinel-2 true-color image chips, each with 224 × 224 pixels and a 512 m × 512 m ground footprint.
Metadata table	CSV	Image filename, five binary infrastructure labels, predefined spatial fold assignment.

The selected bands have a native spatial resolution of 10 m, corresponding to approximately 51 native spatial samples along each side of the 512 m footprint. The footprints were requested directly from the Sentinel Hub Process API as 224 × 224 pixel images, with the default nearest-neighbor upsampling applied. The distributed images therefore provide standardized, model-ready renderings but do not contain spatial information finer than the native 10 m resolution. Nearest-neighbor upsampling may duplicate neighboring pixel values and produce block-like transitions around narrow or high-frequency structures. The 224 × 224 pixel format was selected because it is widely used by pretrained convolutional neural network and Vision Transformer architectures and reduces the need for additional preprocessing for models operating at this input size.

The image collection is designed for multi-label classification rather than mutually exclusive single-class classification. Each image chip is annotated independently for five binary infrastructure labels: electrical substation, power plant, storage tank, railway infrastructure, and major road infrastructure. Electrical substations were derived from OpenStreetMap geometries tagged power=substation, power plants from power=plant, storage tanks from man_made=storage_tank, railway infrastructure from railway=rail and railway=yard, and major road infrastructure from highway=motorway, highway=trunk, and highway=primary [10]. A single image chip may therefore contain no infrastructure label, one positive label, or several positive labels at the same time.

The labels were generated automatically from the retained OpenStreetMap geometries under the specified filtering rules and were not exhaustively manually verified. A representative subset of 600 image chips was subjected to manual visual validation as described in the Experimental Design, Materials and Methods section. Their accuracy may nevertheless be affected by differences in OpenStreetMap completeness, positional accuracy, tagging practices, and temporal alignment with the Sentinel-2 observations.

Table 2 reports the number of positive labels assigned to each image chip. Single-label images account for 58.12% of the collection, while 21.88% contain at least two positive labels. Images with three or more labels represent 3.54% of the dataset, confirming a genuine but imbalanced multi-label setting.

**Table 2:** Distribution of label cardinality across the image collection. The table reports how many image chips contain zero, one, two, three, four, or five positive infrastructure labels.

Number of labels	Number of images	Percentage
0	2000	20.00%
1	5812	58.12%
2	1834	18.34%
3	329	3.29%
4	22	0.22%
5	3	0.03%
Total	10,000	100.00%

Table 3 summarizes label frequencies and pairwise co-occurrences. Diagonal entries indicate the total number of images containing the corresponding label, while off-diagonal entries report the number of images containing both labels. Railway infrastructure and major roads show the most frequent pairwise co-occurrence, followed by substations and railway infrastructure.

**Table 3:** Label frequencies and pairwise co-occurrences.

	Substation	Power plant	Storage tank	Rail	Major road
Substation	2494	241	230	532	432
Power plant	241	1627	148	277	219
Storage tank	230	148	1852	168	151
Rail	532	277	168	2298	585
Major road	432	219	151	585	2299

Images with positive labels are associated with retained OpenStreetMap geometries according to the geometric criteria described in the Experimental Design, Materials and Methods section. No-label images were generated from neighboring candidate locations and accepted only when their complete 512 m × 512 m footprints did not intersect any retained label geometry or overlap previously accepted image footprints. The no-label category therefore denotes the absence of the selected infrastructure categories under the stated OpenStreetMap filtering rules. It does not imply the absence of all infrastructure, built-up areas, roads, rail-related features, or other human-made structures.

**Fig. 1:**
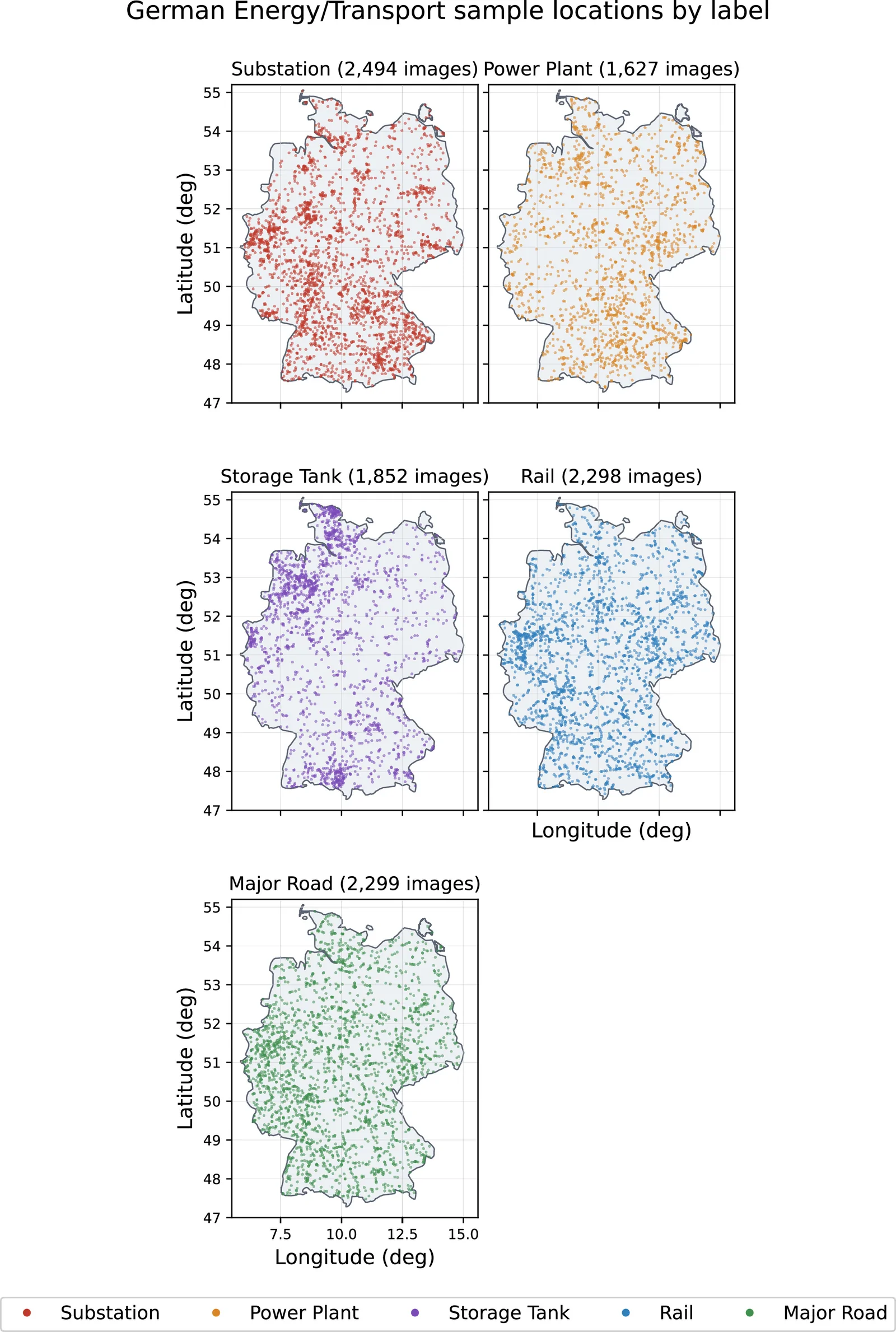
Spatial distribution of accepted sample locations by infrastructure label across Germany. The panels show accepted locations for electrical substations, power plants, storage tanks, railway infrastructure, and major road infrastructure. Each point denotes the WGS84 center coordinate of an image chip associated with the respective label. Sampling was spatially balanced where possible, but the final distributions reflect the unequal frequency and geographic concentration of the underlying OpenStreetMap geometries.

Fig. 1 shows the spatial distribution of accepted sample locations separately for the five infrastructure labels. The sampling procedure was designed to improve spatial balance and reduce excessive clustering in densely mapped regions. However, the final distributions are not expected to be geographically uniform because the underlying infrastructure types are themselves unevenly distributed. Power plants, storage tanks, and major transport infrastructure frequently occur in industrial clusters, along transport corridors, or near particular settlement and land-use structures. The visible concentrations therefore reflect both the balancing procedure and the geographic distribution of the retained OpenStreetMap geometries.

To support reproducible evaluation with reduced spatial leakage, every image was assigned to one of five predefined spatial folds. Internal image-center coordinates were grouped into 20 km spatial blocks. Blocks were merged when samples on opposite block boundaries were within 2 km, while associated no-label samples and samples derived from the same local OpenStreetMap feature were kept together. This procedure produced 764 indivisible spatial groups, none of which was divided across folds.

The five folds contain between 1999 and 2001 images and are closely balanced in terms of individual labels, no-label images, label cardinality, and contextual environments. The minimum center-to-center distance between samples assigned to different folds is 2008 m, and the minimum projected edge-to-edge distance between their image footprints is 1297 m. No image footprints from different folds overlap. The released metadata provides predefined fold identifiers without publishing geographic coordinates. The geographic coordinates and source geometries used during dataset construction were derived from OpenStreetMap geospatial data [10], which are distributed under the Open Database License (ODbL) 1.0. Because redistribution of these OSM-derived geospatial records would require separate consideration of the applicable ODbL attribution and share-alike terms, they are not included in the released metadata under the license applied to the present dataset. The predefined spatial-fold assignments are provided instead, allowing the exact evaluation partitions to be reused without access to the original sampling coordinates.

The metadata table links every image filename to its assigned labels and spatial partition. Its structure is illustrated in Table 4 using an example record.

**Table 4:** Example record from the metadata table.

file_name	substation	power_plant	tank_storage	rail	major_road	spatial_fold
osm_way_878,036,162_rail_sample_1.png	1	0	0	1	0	3

For descriptive purposes, each image chip was assigned a contextual environment label derived from OpenStreetMap area geometries [10]. Intersecting areas were grouped into agriculture or open vegetation, urban, forest, industrial, and water or wetland categories based on selected OpenStreetMap tags. The geometries were clipped to the chip footprint and the overlap area was calculated for each category. The category with the largest overlap was assigned to the chip. Chips without qualifying OpenStreetMap area overlap were assigned the label unknown_osm. Table 5 reports the aggregate distribution of these derived labels across the complete image collection and does not constitute reference land-cover data.

**Table 5:** Distribution of contextual environment labels across the image collection.

Environment label	Number of images	Percentage of dataset
agriculture_or_open_vegetation	4692	46.92
urban	2966	29.66
forest	1457	14.57
industrial	812	8.12
water_or_wetland	56	0.56
unknown_osm	17	0.17
Total	10,000	100.00

Agricultural or open vegetation and urban environments together account for 76.58% of the collection, while industrial and water-related contexts are less frequent. These distributions reflect the environments surrounding the sampled infrastructure and should be considered when interpreting model performance across contextual conditions.

**Fig. 2:**
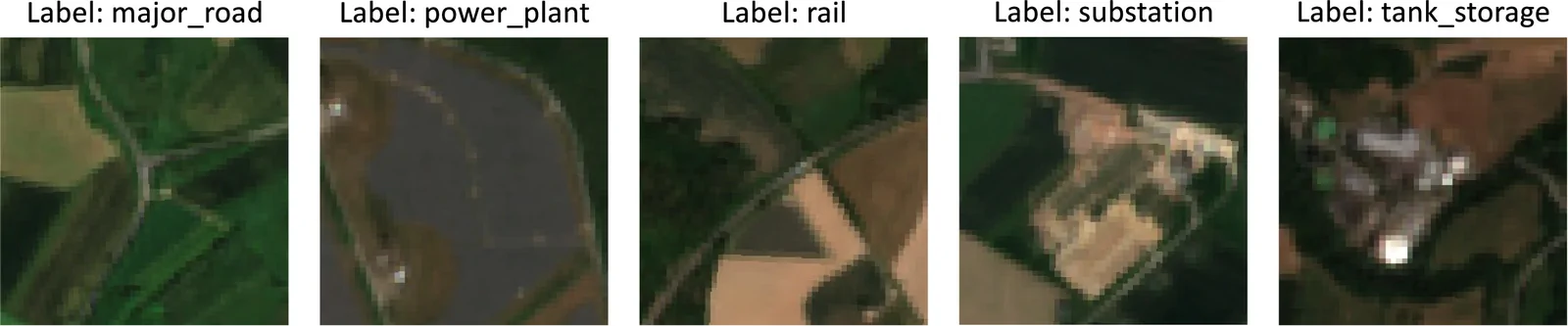
Example Sentinel-2 image chips from the dataset. The panels show selected major_road, power_plant, rail, substation, and tank_storage image chips across several contextual environment labels. Images are rendered from Sentinel-2 Level-2A B04, B03, and B02 bands [7,9] and cover 512 m × 512 m footprints.

Fig. 2 presents selected image chips from all infrastructure classes. The figure illustrates the visual form, environmental diversity, and spatial extent of the released image data. Each panel shows one 224 × 224 pixel RGB image representing a 512 m × 512 m footprint.

To quantitatively describe visual variation in the image collection, four image-level characteristics were calculated for each RGB chip and summarized overall and separately for all labels in Table 6. Brightness denotes the mean normalized Value component in the HSV color space, whereas saturation denotes the mean normalized HSV Saturation component. Michelson contrast quantifies the grayscale intensity range using the first and 99th percentiles, reducing sensitivity to extreme pixel values [11]. Canny edge density represents the proportion of pixels identified as edges after grayscale conversion and Gaussian smoothing [12].

**Table 6:** Descriptive image statistics (Average, Avg., and Standard Deviation, SD) for the complete collection and all labels.

Label	Brightness		Saturation		Michelson Contrast		Canny Edge Density	
	Avg.	SD	Avg.	SD	Avg.	SD	Avg.	SD
*Overall*	0.2078	0.0628	0.4219	0.0732	0.7007	0.1087	0.0090	0.0119
substation	0.2169	0.0542	0.3984	0.0689	0.7184	0.0934	0.0123	0.0134
power_plant	0.2190	0.0594	0.3970	0.0767	0.7179	0.1153	0.0107	0.0118
tank_storage	0.2156	0.0623	0.4333	0.0687	0.7084	0.0987	0.0099	0.0129
rail	0.2121	0.0645	0.4020	0.0763	0.7185	0.1063	0.0123	0.0148
major_road	0.2055	0.0602	0.4126	0.0684	0.7137	0.0976	0.0096	0.0120
no label	0.1967	0.0728	0.4522	0.0700	0.6681	0.1256	0.0055	0.0099

The statistics indicate moderate differences in global visual characteristics across most infrastructure labels. This suggests that the classes cannot be distinguished solely through brightness, saturation, contrast, or edge density, although these statistics do not exclude the use of other contextual or dataset-specific cues by trained models. No-label chips show slightly lower average brightness, contrast, and edge density but higher saturation than the complete collection, which is consistent with their selection outside the retained infrastructure geometries.

## 4 Experimental Design, Materials and Methods

The dataset was generated through a reproducible workflow consisting of five main steps: extraction of infrastructure reference geometries from OpenStreetMap [10], spatially balanced candidate selection across Germany, multi-label assignment based on footprint-level geometry intersections, retrieval of corresponding Sentinel-2 image chips, and construction of predefined spatial cross-validation folds. Implementation-independent pseudocode for the principal data-generation procedures is provided in Appendix A (Algorithms A.1–A.5).

Recent publications demonstrate the use of Sentinel-2 imagery for reusable machine-learning datasets, including OpenStreetMap-based road-detection masks, multimodal Sentinel-1 and Sentinel-2 image collections, and cloud and cloud-shadow classification benchmarks [[4], [5], [6]]. These datasets show how transparent acquisition procedures and task-specific labels can support reproducible remote sensing research. However, many infrastructure-oriented Sentinel-2 datasets focus on individual object categories or mutually exclusive labels. The present workflow addresses this limitation by constructing a multi-label image collection in which several energy, transport, and storage infrastructure categories may occur within the same 512 m × 512 m footprint.

### 4.1 Infrastructure geometry extraction and candidate selection

Infrastructure geometries and contextual land-cover information were derived from an OpenStreetMap extract covering Germany [10]. The extract was processed locally using pyosmium [13]. Five infrastructure labels were retained: electrical substation, power plant, storage tank, railway infrastructure, and major road infrastructure. Electrical substations were derived from geometries tagged power=substation, power plants from power=plant, storage tanks from man_made=storage_tank, railway infrastructure from railway=rail and railway=yard, and major road infrastructure from highway=motorway, highway=trunk, and highway=primary. The retained features were used both as candidate sources and as indexed reference geometries for footprint-level intersection tests.

Candidate points for linear infrastructure were generated at the start and end of each retained geometry and at intervals of 2500 m along its course. For polygonal infrastructure, the polygon centroid was used when it lay inside the exterior ring. Otherwise, the first coordinate of the exterior ring was retained. After filtering to the German study extent, 1294,335 source geometries remained.

To reduce excessive concentration in densely mapped infrastructure regions, the study extent was divided into a dynamically generated grid containing 1235 × 1049 cells. Candidate sources were ordered through a deterministic SHA-256-based spatial round-robin procedure across occupied cells and subsequently limited to 50,000 sources. A 512 m × 512 m footprint was constructed around each candidate center, and candidates were accepted sequentially only when their footprints satisfied the applicable geometry-intersection and non-overlap conditions.

### 4.2 Multi-label assignment and no-label sampling

Unlike a single-label classification dataset, the present dataset does not assign each image to exactly one class. Each accepted footprint was tested independently against the retained geometries of all five infrastructure categories. For image iand class c, the binary label was defined as

(1) $$y_{i,c}=I\left(\exists g\in G_{c}:F_{i}\cap g\neq \emptyset \right)$$

where $F_{i}$denotes the footprint of image i, $G_{c}$is the set of retained OpenStreetMap geometries for class c, and Iis the indicator function. A chip may therefore receive no positive label, one positive label, or several positive labels simultaneously.

No-label samples were generated at neighboring locations around retained infrastructure candidates. Candidate centers were examined in concentric rings with increments of 512 m, a maximum of 36 rings, and a fixed directional order. A no-label candidate was accepted only when its complete footprint remained within the study extent, did not overlap a previously accepted footprint, and did not intersect any retained geometry belonging to the five infrastructure definitions. The intersection test was performed against the complete indexed geometries rather than candidate points alone.

The resulting no-label samples had anchor distances between 512 m and 3620 m. Of the 2000 accepted no-label samples, 1939 were obtained from the first search ring. Their local proximity to infrastructure candidates provides contextually similar negative examples while the footprint-level exclusion rules prevent intersections with the selected target geometries. The no-label annotation denotes only the absence of the five retained OpenStreetMap categories and does not imply the absence of all infrastructure, roads, buildings, rail-related features, or other human-made structures.

### 4.3 Sentinel-2 image acquisition and resampling

For every accepted location, a 512 m × 512 m image chip was retrieved from the Copernicus Sentinel-2 Level-2A collection through the Sentinel Hub Process API of the Copernicus Data Space Ecosystem [14]. Sentinel-2 is an optical Earth-observation mission for systematic land and coastal monitoring. The satellites operate in a near-polar, sun-synchronous orbit at an altitude of approximately 786 km and provide a nominal revisit interval of about five days at the equator. During the acquisition period, the available collection included observations from Sentinel-2A, Sentinel-2B, and Sentinel-2C [7,9].

Each platform carries the MultiSpectral Instrument, a push-broom sensor with a 290 km swath that records 13 spectral bands between 443 and 2190 nm. Four bands are provided at 10 m, six at 20 m, and three at 60 m spatial resolution. The instrument uses 12-bit radiometric quantization, and Level-2A products provide atmospherically corrected bottom-of-atmosphere surface reflectance. For this dataset, the 10 m bands B04, B03, and B02 were used as the red, green, and blue channels, respectively [7,9].

Imagery was requested for the period from 06 July 2025 to 05 July 2026 using a maximum cloud-cover threshold of 10% and the leastCC mosaicking option [14]. Each 512 m footprint was requested directly as a 224 × 224 pixel PNG image, corresponding to an output spacing of approximately 2.286 m per rendered pixel. This value does not represent the physical spatial resolution. The underlying imagery remains limited to the native 10 m resolution of the selected bands, corresponding to approximately 51 native spatial samples along each side of the footprint.

No explicit upsampling method was specified in the Process API request, so the Sentinel-2 Level-2A default nearest-neighbor method was applied [14]. The returned PNG files were stored without further rescaling during dataset export. Nearest-neighbor upsampling may repeat native pixel values and introduce block-like transitions around narrow or high-frequency structures. The 224 × 224 representation therefore standardizes the input dimensions for computer vision applications without adding spatial information beyond the original Sentinel-2 observations.

The export workflow used authenticated API requests, bounded parallel processing, retry handling for temporary failures, PNG validation, and resumable execution. All 10,000 image chips were successfully generated and verified to have dimensions of 224 × 224 pixels. A detailed implementation-independent representation of the Sentinel-2 acquisition and export workflow, including the Process API configuration and request handling, is provided in Algorithm A.5.

### 4.4 Contextual environment labels

Each chip was additionally assigned a descriptive environment label based on intersecting OpenStreetMap area geometries [10]. Selected landuse, natural, building, amenity, aeroway, and man_made tags were grouped into the categories industrial, urban, forest, water or wetland, and agriculture or open vegetation. Each intersecting geometry was clipped to the rectangular image footprint. For a polygon exterior ring with vertices $\left(x_{1},y_{1}),\ldots ,(x_{m},y_{m}\right)$, the coordinate-area score was calculated using the shoelace formula

(2) $$A\left(P\right)=\frac{1}{2}\left|\sum_{r=1}^{m}\left(x_{r}y_{r+1}-x_{r+1}y_{r}\right)\right|,\;\left(x_{m+1},y_{m+1}\right)=\left(x_{1},y_{1}\right)$$

The geometries were processed in WGS84 coordinates. MultiPolygon exterior rings were evaluated separately and summed. Interior rings were not subtracted and overlapping geometries within the same category were not geometrically merged. The resulting values are therefore relative overlap scores in squared coordinate units rather than physical area estimates.

These environment labels are intended only as descriptive contextual variables. They are not reference land-cover annotations, and their interpretation is limited by OpenStreetMap completeness, overlapping geometries, omitted polygon holes, and the use of coordinate-based rather than metric area scores.

### 4.5 Spatial fold construction

To reduce spatial leakage during model evaluation, each image was assigned to one of five predefined spatial folds. The coordinates were initially grouped into square 20 km blocks. Blocks were merged when samples located on opposite block boundaries had center-to-center distances of at most 2 km. No-label samples were also kept together with their source-candidate anchors, and samples associated with the same local OpenStreetMap feature were assigned to the same spatial group.

This procedure produced 952 initial grid blocks and 764 final indivisible spatial groups. The groups were assigned to five folds using 256 deterministic greedy restarts with seed 20,260,802. For feature jand fold k, the assignment objective was

(3) $$J=\sum_{j}w_{j\sum_{k=1}^{K}{\left(\frac{N_{k,j}-\mu_{j}}{\max\left(\mu_{j},1\right)}\right)}^{2}},\;\;\mu_{j}=\frac{N_{j}}{K}$$

where $N_{k,j}$is the value of balancing feature jassigned to fold k, $N_{j}$is its dataset-wide total, and K=5. The feature weights were 3.0 for fold size, 1.5 for each infrastructure label and the no-label category, 0.75 for label-cardinality groups containing one, two, or at least three labels, and 0.25 for each environment category. Spatial grouping always had priority over numerical balance.

The resulting folds contain between 1999 and 2001 images and closely matched class, no-label, label-cardinality, and environment distributions. No spatial group was divided between folds. The minimum center-to-center distance between samples assigned to different folds was 2008 m, and the minimum projected edge-to-edge distance between their footprints was 1297 m. No footprints assigned to different folds overlapped.

The released metadata contains the predefined fold assignment. This allows identical outer cross-validation folds to be reused independently of access to the original sampling coordinates.

### 4.6 Manual visual validation of openstreetmap-derived labels

To assess the visual consistency of the OpenStreetMap-derived annotations, a representative subset of 600 image chips was manually reviewed. The subset was selected using a random stratified sampling procedure, with equal representation of the five predefined spatial folds. Sampling additionally aimed to preserve the distributions of the infrastructure labels, no-label samples, label cardinality, and frequent label combinations observed in the complete dataset. For each image chip, all assigned and absent labels were systematically compared with the infrastructure identifiable in the corresponding Sentinel-2 RGB image.

The manual review did not identify any visually evident mislabeled images or cases in which an annotation contradicted the corresponding image content. These findings support the overall visual consistency and reliability of the OpenStreetMap-derived annotations across the representative validation subset.

### 4.7 Baseline image-classification evaluation

To provide an initial reference benchmark, four established image-classification backbones were evaluated: ResNet-50 [15], Swin Transformer V2 Base [16], EfficientNet-B4 [17], and ShuffleNet V2 [18]. The selected models represent convolutional and transformer-based architectures with different capacities and computational requirements.

The original classification head of each backbone was replaced by a two-layer classification head comprising a 512-unit hidden layer, ReLU activation, dropout of 0.2, and a five-unit output layer corresponding to electrical substation, power plant, storage tank, railway infrastructure, and major road infrastructure. Independent logits were converted to label probabilities using sigmoid activation. All backbones were initialized with ImageNet-1K-pretrained weights and trained in two stages using AdamW [19]. First, the backbone was frozen and the classification head was trained for up to 10 epochs with a learning rate of $3\;\times 10^{-4}$. Subsequently, the complete model was fine-tuned for up to 25 epochs using architecture-specific learning rates of 3.0e-5 for ResNet-50, 1.0e-5 for Swin Transformer V2 Base, 2.0e-5 for EfficientNet-B4, and 3.0e-5 for ShuffleNet V2. Weight decay was set to $10^{-4}$, gradients were clipped to a global norm of 1.0, and the effective batch size was 32. A one-epoch linear warm-up was followed by cosine annealing [20]. Early stopping was applied in both stages, and the checkpoint with the lowest internal validation BCE loss was retained.

Model performance was evaluated using the five predefined spatial folds. In each outer run, one complete fold containing approximately 2000 images was held out for testing. From the remaining four folds, approximately 800 images were selected through a deterministic multi-label-stratified procedure as an internal validation subset, while approximately 7200 images were used for training. The outer spatial test fold was not used for loss weighting, checkpoint selection, early stopping, threshold calibration, or model selection.

Class imbalance was addressed using a weighted binary cross-entropy loss. For a batch containing Bimages and C=5labels, the loss was defined as

(4) $$L_{BCE}=-\frac{1}{BC}\sum_{i=1}^{B}\sum_{c=1}^{C}\left[w_{c}^{+}y_{i,c}\log\left(p_{i,c}\right)+\left(1-y_{i,c}\right)\log\left(1-p_{i,c}\right)\right]$$

where $p_{i,c}=\sigma \left(z_{i,c}\right)$is the sigmoid probability derived from logit $z_{i,c}$. Positive-class weights were calculated only from the training subset as

(5) $$w_{c}^{+}=\min\left(\frac{N_{c}^{-}}{N_{c}^{+}},20\right)$$

where $N_{c}^{+}$and $N_{c}^{-}$denote the numbers of positive and negative training samples for label c, respectively. For each architecture and outer fold, the checkpoint with the lowest internal validation BCE loss was retained for evaluation on the held-out spatial test fold.

Predictions were evaluated using both a fixed decision threshold of 0.5 and label-specific calibrated thresholds. For each outer fold, the calibrated threshold of every label was selected exclusively from the internal validation predictions by maximizing the corresponding binary F1 score. Thresholds between 0.05 and 0.95 were examined in increments of 0.01. In the event of a tie, the threshold closest to 0.5 was retained. The selected thresholds were then applied unchanged to the corresponding outer spatial test fold. Implementation-independent pseudocode for the spatial cross-validation and two-stage training procedure is provided in Algorithm A.6, while threshold calibration and out-of-fold evaluation are detailed in Algorithm A.7.

Performance was assessed using accuracy, balanced accuracy, precision, recall, and F1 score. To provide a more comprehensive evaluation of the multi-label setting, exact-match accuracy, micro-averaged F1 score, and macro average precision (mAP) were additionally calculated. The former two complement the label-wise macro metrics with strict sample-level and globally pooled multi-label evaluations, respectively, while mAP provides a threshold-independent assessment based on the predicted label probabilities.

For label c, let $TP_{c}$, $TN_{c}$, $FP_{c}$, and $FN_{c}$denote the numbers of true positives, true negatives, false positives, and false negatives, respectively. The label-wise metrics were calculated as follows:

(6) $$\text{Accurac}y_{c}=\frac{TP_{c}+TN_{c}}{TP_{c}+TN_{c}+FP_{c}+FN_{c}}$$

(7) $$\text{Balanced}\;\text{Accurac}y_{c}=\frac{1}{2}\left(\frac{TP_{c}}{TP_{c}+FN_{c}}+\frac{TN_{c}}{TN_{c}+FP_{c}}\right)$$

(8) $$\text{Precisio}n_{c}=\frac{TP_{c}}{TP_{c}+FP_{c}}$$

(9) $$\text{Recal}l_{c}=\frac{TP_{c}}{TP_{c}+FN_{c}}$$

(10) $$F1_{c}=\frac{2\;\text{Precisio}n_{c}\;\text{Recal}l_{c}}{\text{Precisio}n_{c}+\text{Recal}l_{c}}$$

Each metric was macro-averaged across the five infrastructure labels so that every label contributed equally, irrespective of its prevalence:

(11) $$M_{\text{macro}}=\frac{1}{C}\sum_{c=1}^{C}M_{c},\;\;C=5$$

For the complementary multi-label metrics, N denotes the number of images and L the number of labels, with L equal to 5. For each image and label, the reference annotation and the corresponding binary prediction can each take a value of either 0 or 1. Exact-match accuracy was calculated as the proportion of images for which the complete five-label prediction vector matched the corresponding reference annotation:

(12) $$\text{ExactMatch}=\frac{1}{N}\sum_{i=1}^{N}1\left(y_{i}=\hat{y_{i}}\right)$$

For micro-F1, true positives, false positives, and false negatives were pooled across all images and labels:

(13) $$F1_{\text{micro}}=\frac{2TP_{\text{micro}}}{2TP_{\text{micro}}+FP_{\text{micro}}+FN_{\text{micro}}}$$

Average precision was calculated separately for each label from the ranked predicted probabilities and the corresponding precision-recall relationship. Macro average precision was then obtained by averaging the label-wise average precision values:

(14) $$\text{mAP}=\frac{1}{L}\sum_{l=1}^{L}AP_{l}$$

Exact-match accuracy and micro-F1 were calculated from the calibrated binary predictions, whereas mAP was calculated directly from the predicted probabilities and is therefore independent of the selected decision thresholds.

Table 7, Table 8 report the mean and standard deviation across the five spatial test folds for all evaluated backbones. Additional label-wise numerical results based on the pooled out-of-fold predictions are provided in Appendix B (Table B.1).

**Table 7:** Calibrated multi-label performance across five spatial test folds. Values are reported as mean ± standard deviation.

Backbone	Macro Accuracy	Macro Bal. Acc.	Macro Precision	Macro Recall	Macro F1 score
ResNet-50 [15]	0.8290 ± 0.0083	0.7655 ± 0.0070	0.6083 ± 0.0160	0.6598 ± 0.0200	0.6252 ± 0.0107
Swin V2 Base [16]	0.8498 ± 0.0082	0.7931 ± 0.0028	0.6510 ± 0.0218	0.6975 ± 0.0107	0.6689 ± 0.0080
EfficientNet-B4 [17]	0.8047 ± 0.0154	0.7462 ± 0.0078	0.5577 ± 0.0426	0.6513 ± 0.0443	0.5908 ± 0.0089
ShuffleNet V2 [18]	0.8105 ± 0.0087	0.7526 ± 0.0102	0.5741 ± 0.0212	0.6580 ± 0.0273	0.6020 ± 0.0135

**Table 8:** Complementary multi-label performance across five spatial test folds. Values are reported as mean ± standard deviation.

Backbone	Exact-match accuracy	Micro F1 score	mAP
ResNet-50 [15]	0.4323 ± 0.0256	0.6217 ± 0.0101	0.6784 ± 0.0064
Swin V2 Base [16]	0.4785 ± 0.0299	0.6631 ± 0.0087	0.7265 ± 0.0114
EfficientNet-B4 [17]	0.3651 ± 0.0340	0.5879 ± 0.0086	0.6257 ± 0.0107
ShuffleNet V2 [18]	0.3655 ± 0.0198	0.5969 ± 0.0133	0.6414 ± 0.0143

Swin Transformer V2 Base achieved the strongest mean performance for all reported metrics. It reached a macro accuracy of 0.8498 ± 0.0082, a macro balanced accuracy of 0.7931 ± 0.0028, and a macro F1 score of 0.6689 ± 0.0080. The complementary multi-label metrics showed the same overall pattern, with Swin Transformer V2 Base achieving an exact-match accuracy of 0.4785 ± 0.0299, a micro-F1 score of 0.6631 ± 0.0087, and a macro average precision of 0.7265 ± 0.0114. ResNet-50 achieved the second-highest performance, followed by ShuffleNet V2 and EfficientNet-B4. The comparatively small standard deviations indicate that the relative performance was stable across the five spatial test folds. These baseline results are intended as reference values for future use of the dataset and should not be interpreted as an independent measure of annotation quality, as model performance may also reflect label noise, spatial context, and the spectral and spatial limitations of the Sentinel-2 RGB imagery.

For Swin Transformer V2 Base, the selected fine-tuning checkpoints occurred between epochs 13 and 18. The corresponding training losses ranged from 0.57859 to 0.61821, while the internal validation losses ranged from 0.63,993 to 0.68,113. Together with the observed recall and F1 scores, these fold-wise results indicate stable convergence and show that the model did not collapse toward almost exclusively negative predictions.

Table 9 reports the label-wise performance of Swin Transformer V2 Base based on the pooled outer test predictions. Railway infrastructure achieved the highest F1 score, closely followed by major roads and power plants. Electrical substations remained the most challenging category, particularly in terms of precision and balanced accuracy.

**Table 9:** Label-wise calibrated performance of Swin Transformer V2 Base based on pooled out-of-fold test predictions.

Label	Precision	Recall	F1 score	Bal. Acc.
Major road	0.6849	0.7234	0.7036	0.8120
Power plant	0.7190	0.6620	0.6893	0.8058
Rail	0.7243	0.7032	0.7136	0.8117
Substation	0.5065	0.6836	0.5819	0.7312
Storage tank	0.6053	0.7154	0.6558	0.8047

**Fig. 3:**
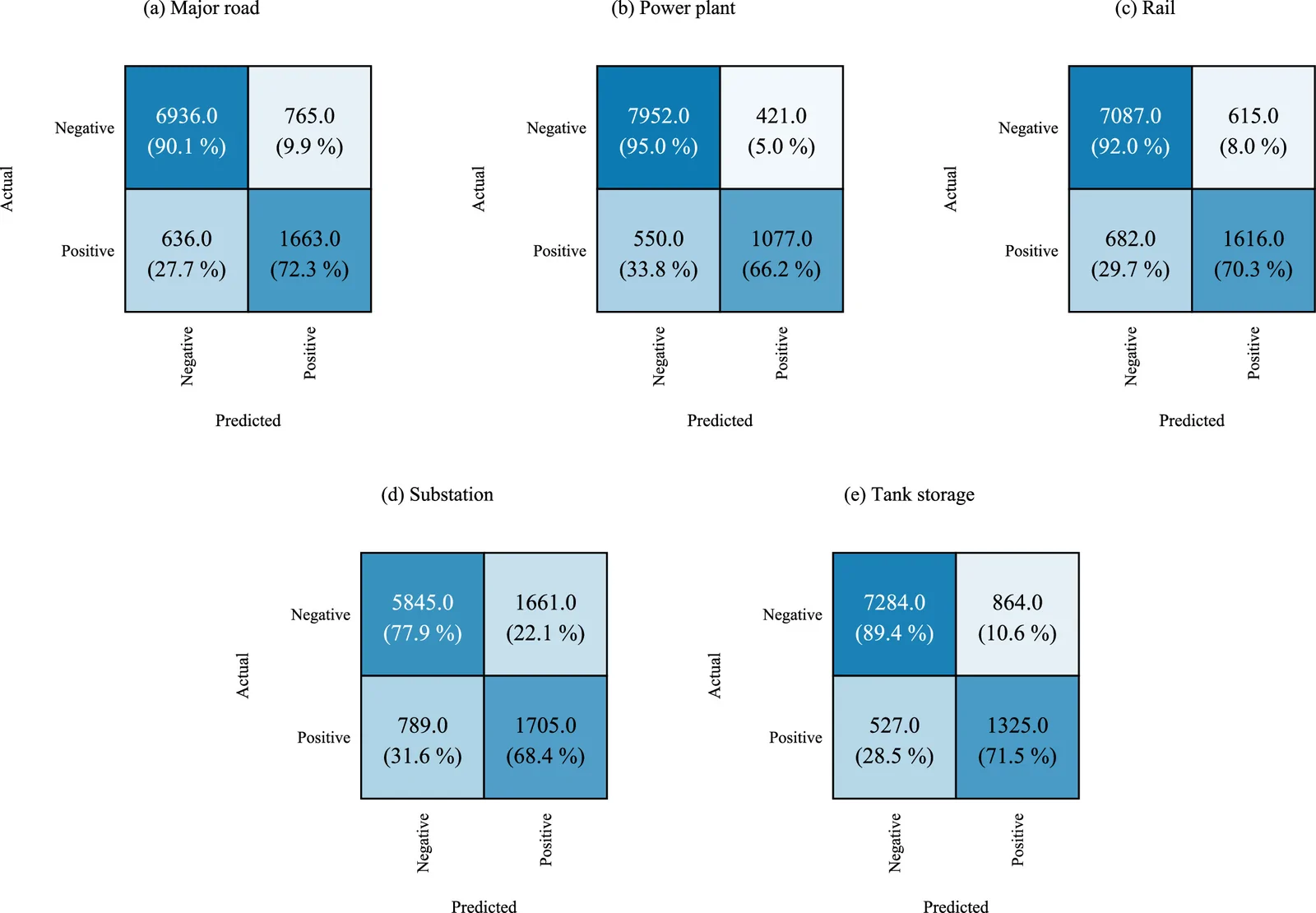
Label-wise confusion matrices for Swin Transformer V2 Base aggregated from the outer test predictions of all five spatial folds using the calibrated thresholds. Separate binary matrices are shown for substation, power_plant, tank_storage, rail, and major_road. Percentages are reported relative to the true state of the respective label.

Fig. 3 presents the label-wise confusion matrices for Swin Transformer V2 Base, aggregated from the outer test predictions of all five folds. Because the task is multi-label rather than mutually exclusive, a separate binary confusion matrix is shown for each label. Each matrix reports true negatives, false positives, false negatives, and true positives for the respective infrastructure category. The corresponding numerical confusion-matrix counts are provided in Appendix B (Table B.1).

To further support independent reproduction of the workflow, Appendix A provides implementation-independent pseudocode for the principal dataset-generation, Sentinel-2 acquisition, spatial-partitioning, model-training, threshold-calibration, and evaluation procedures. Appendix B additionally provides detailed numerical baseline results and the numerical values underlying the reported confusion matrices.

## Limitations

The infrastructure labels are derived from a specific OpenStreetMap extract [10] and therefore depend on the completeness, positional accuracy, temporal status, and tagging practices of the source data. Temporal differences between the OpenStreetMap extract and the Sentinel-2 observations may introduce additional inconsistencies. A negative label indicates only that the complete chip footprint did not intersect a retained OpenStreetMap geometry for that category after filtering. It does not prove the absence of infrastructure that may be missing, differently tagged, inaccurately positioned, or outside the dataset definitions. The no-label images should be interpreted accordingly and may still contain buildings, minor roads, industrial areas, or other human-made structures.

The image chips contain only the Sentinel-2 true-color bands B02, B03, and B04, each with a native spatial resolution of 10 m [9]. Accordingly, a 512 m × 512 m footprint contains only approximately 51 native spatial samples along each image axis. Small or narrow infrastructure elements may therefore occupy only a few native pixels or be represented by mixed pixels containing both infrastructure and surrounding land cover. The 224 × 224 pixel images are nearest-neighbor-resampled renderings of these observations. This resampling increases the image dimensions for model compatibility but does not recover additional spatial detail and may introduce repeated pixel blocks and block-like transitions. Consequently, the visual identification of small or ambiguous infrastructure may remain difficult, and classification models may partly rely on broader spatial and land-use context rather than fine-grained object morphology. In addition, restricting the imagery to the three visible bands excludes near-infrared, red-edge, and shortwave-infrared information that could provide additional spectral cues for distinguishing infrastructure from vegetation, soil, and other surrounding materials.

The imagery was acquired over a one-year period and may originate from different acquisition dates and Sentinel-2 platforms. Seasonal conditions, illumination, atmospheric effects, and land-surface changes may therefore vary between image chips. Finally, the dataset covers Germany only, so its geographic scope, infrastructure definitions, sampling strategy, and acquisition period should be considered when transferring models to other regions or time periods.

## Ethics Statement

The authors have read and follow the ethical requirements for publication in Data in Brief. The present work does not involve human participants, animal experiments, or data collected from social media platforms. The dataset was generated from publicly available OpenStreetMap data [10] and Copernicus Sentinel-2 satellite imagery [7,9].

## Credit Author Statement

**Lukas Pasold:** Software, Methodology, Formal analysis, Investigation, Data Curation, Visualization, Writing – Original Draft. **Luca Eisentraut:** Formal analysis, Methodology, Validation, Investigation, Writing – Original Draft. **Felix Waigner:** Software, Methodology, Validation, Investigation, Writing – Original Draft. **Ricardo Buettner:** Conceptualization, Funding acquisition, Methodology, Project administration, Supervision, Writing – Review & Editing.

## Data Availability

ZenodoA Multi-Label Sentinel-2 Dataset for Deep Learning-Based Energy, Transport, and Storage Infrastructure Mapping in Germany (Original data)

## Appendix Supplementary materials

Image, application 1
