# Supplementary material for: A multi-label sentinel-2 dataset for deep learning-based energy, transport, and storage infrastructure mapping in Germany
Source: Data Brief. 2026 Sep 3;69:113226. doi: 10.1016/j.dib.2026.113226 (PMC13584112; doi:10.1016/j.dib.2026.113226)
Supplement: Supplementary file 1 [file mmc1.docx]

# APPENDIX A. PSEUDOCODE FOR DATA GENERATION AND EVALUATION

## Algorithm A.1: OpenStreetMap Geometry Extraction and Filtering

**Input:**
OpenStreetMap data

**Output:**
Spatially indexed infrastructure geometries for five labels

1. Read the OSM data with node locations enabled and retain objects containing at least one of the keys power, man_made, railway, or highway.
2. Assign labels using the following rules:
   - substation: power = substation
   - power plant: power = plant
   - storage tank: man_made = storage_tank
   - railway infrastructure: railway = rail or railway = yard
   - major road infrastructure: highway ∈ {motorway, trunk, primary}
3. Retain labeled nodes with valid coordinates.
4. Convert labeled OSM areas from ways or relations into exterior-ring polygons. Inner rings are not retained.
5. Represent railway and road ways as individual line segments.
6. Mark a way as a tunnel when tunnel is present and not equal to no, false, 0, or an empty value.
7. Store tunnel segments for reference but exclude them from candidate generation and later line-intersection labels.
8. Store points, polygons, and line segments in separate spatial tables with R-tree bounding-box indexes.
9. For non-tunnel lines, additionally generate candidate source points at the first vertex, every 2,500 m along the line, and the final vertex.
10. Store only selected descriptive OSM tags. Storage tanks are retained regardless of their content tag.

## Algorithm A.2: Spatially Balanced Candidate Selection and Multi-Label Assignment

**Input:**
Indexed OSM geometries, Germany boundary, footprint size 512 m

**Output:**
8,000 non-overlapping positive samples with five binary labels

1. Create source candidates from:
   - retained OSM point locations;
   - one representative point per polygon, using the centroid if it lies inside the polygon and otherwise the first polygon vertex;
   - line samples spaced at 2,500 m.
2. Retain a candidate if its center lies inside Germany.
3. Construct a 512 m × 512 m footprint around every center using local meter-to-degree conversion.
4. Spatially distribute candidates over a country-wide grid and deterministically interleave candidates from the occupied grid cells.
5. Retain at most the first 50,000 spatially ordered source candidates.
6. In the default fast mode, initially group candidates by their source label. Retain at most 2,500 candidates per preliminary label combination.
7. First request up to 500 candidates from every available combination. Then add candidates iteratively from the currently least represented combinations until 8,000 positives are selected.
8. Reject a candidate if its footprint overlaps any previously accepted footprint. Footprints that only touch at their boundaries are permitted.
9. After selection, recompute all five labels for every footprint:
   - point labels are assigned when a point lies inside the footprint;
   - polygon labels are assigned when the polygon intersects the footprint;
   - rail and road labels are assigned when a non-tunnel line segment intersects the footprint.
10. Store the labels in the fixed order substation, power plant, storage tank, railway, and major road.
11. Abort if fewer than 8,000 positive samples are obtained or if final validation detects an overlap or label mismatch.

## Algorithm A.3: No-Label Sample Generation

**Input:**
8,000 accepted positive samples and the indexed OSM geometries

**Output:**
2,000 nearby samples without any of the five labels

1. Sort the positive samples deterministically using a hash of their candidate identifiers.
2. For each positive sample, search for at most one nearby no-label sample.
3. Examine search rings 1–36 at multiples of the 512 m footprint size.
4. Within each ring, test the following directions in fixed order: west, east, south, north, southwest, northwest, southeast, and northeast.
5. Reject a candidate if:
   - its center lies outside Germany;
   - its footprint overlaps a positive or previously accepted no-label footprint;
   - its spatial uniqueness cell has already been used; or
   - its complete footprint intersects any retained geometry belonging to the five infrastructure labels.
6. The intersection test includes all relevant points and polygons and all non-tunnel railway and major-road segments.
7. Accept the first valid candidate found for the current positive sample and record its positive anchor and offset.
8. Stop when 2,000 no-label samples have been accepted or all positive samples have been searched.
9. Abort unless exactly 2,000 no-label samples are obtained.
10. Validate that all 10,000 final footprints are mutually non-overlapping and that every no-label footprint has an empty five-label vector.

## Algorithm A.4: Spatial Fold Construction

**Input:**
10,000 samples with coordinates, labels, source relationships, OSM identifiers, and environment categories

**Output:**
Five spatial folds with indivisible spatial groups

1. Project all sample centers to ETRS89 / LAEA Europe (EPSG:3035).
2. Assign each sample to a square 20 km grid block.
3. Form spatial groups by transitively merging:
   - all samples in the same 20 km block;
   - every no-label sample with its positive source sample;
   - all samples derived from the same OSM object, identified by OSM type and OSM identifier;
   - samples in different blocks whose centers are at most 2 km apart.
4. Treat every resulting spatial group as indivisible. A group must never be assigned to more than one fold.
5. Represent each group by counts for:
   - total samples;
   - each of the five labels;
   - no-label samples;
   - samples with one, two, or at least three labels;
   - each environment category.
6. Assign complete groups to five folds by minimizing normalized squared deviations from equal per-fold targets.
7. Use weights of 3.0 for total count, 1.5 for each label and the no-label count, 0.75 for label-cardinality counts, and 0.25 for environment counts.
8. Perform 256 deterministic greedy restarts with seed 20,260,802.
9. After each greedy assignment, perform at most 12 improving single-group moves.
10. Retain the assignment with the lowest objective value.
11. Spatial grouping constraints always take priority over numerical balance.
12. For nested cross-validation, divide the non-test spatial groups into ten balanced bins using 16 restarts and four improvement passes; use one bin for validation and nine for training.

## Algorithm A.5: Sentinel-2 Image Acquisition and Export

**Input:**
10,000 sample centers, Copernicus credentials, and image-query settings

**Output:**
One 224 × 224 PNG per successfully processed sample

1. Authenticate against the Copernicus Data Space Ecosystem using OAuth 2.0 client credentials.
2. Verify that exactly 10,000 candidates are present and that all specify a 512 m footprint.
3. For every candidate, recompute a geographic bounding box corresponding to 512 m × 512 m around its center.
4. Submit a direct Sentinel Hub Process API request using:
   - collection: Sentinel-2 Level-2A;
   - period: 6 July 2025 to 5 July 2026;
   - maximum cloud coverage: 10%;
   - mosaicking order: least cloud cover;
   - output size: 224 × 224 pixels;
   - output format: PNG.
5. Request bands B02, B03, B04, and dataMask.
6. Produce the output channels as:
   - red = 2.5 × B04;
   - green = 2.5 × B03;
   - blue = 2.5 × B02;
   - fourth channel = dataMask.
7. No separate STAC scene selection is performed. The complete date range is passed directly to the Process API.
8. No explicit resampling method is specified. Although 10 m resolution is recorded in the settings, it is not included in the request payload.
9. Process candidates in batches of 200 with default concurrency 2. A shared request gate serializes provider requests and applies a default minimum interval of 1 s.
10. Allow eight request attempts by default. Retry authentication failures, transport errors, HTTP 429 responses, and server errors according to the implemented backoff rules.
11. Validate responses by checking the PNG file signature.
12. Store successful images as candidate_identifier.png and append their file names and five labels to the metadata CSV.
13. Record failed candidates in a separate error CSV.
14. On restart, skip candidates already listed in the image CSV. Existing image files without a corresponding CSV entry are not considered completed.

## Algorithm A.6: Multi-Label Classification Protocol (Splits and Two-Stage Training)

**Input:**
ETS dataset root with images/ and images.csv (10,000 rows), local ImageNet backbone weights, configuration with seed 42
**Output:**
Five leakage-checked fold splits and 20 trained model instances (four architectures × five folds)

1. Read the metadata CSV and resolve the image, label and fold columns from configuration or column aliases.
2. Adopt the five binary columns as the label vocabulary: substation, power plant, storage tank, railway, and major road.
3. Reject non-binary or empty label cells, duplicated or missing image files, and fold values outside 1 to 5.
4. Treat an empty label row as the explicit no-label case, never as an additional class.
5. Sort the labels alphabetically and store them as a fixed 10,000 × 5 indicator matrix.
6. Use the predefined spatial folds as outer test folds. Every pipeline fold tests exactly one spatial fold.
7. Divide the remaining pool of each fold into ten label-balanced partitions and use one as validation. Balancing minimizes squared deviations from equal label counts and partition size; each image forms its own group.
8. Verify that train, validation and test are disjoint, then write the splits, the fold manifest and a split report. Each fold holds roughly 7,200 training, 800 validation and 2,000 test images.
9. Perform steps 10 to 15 independently per architecture and fold, seeded with 42 + fold and with deterministic kernels.
10. Build the backbone without its classifier plus a shared head with 512 units, dropout 0.20 and five outputs, and load the local ImageNet weights.
11. Augment training images with random resized crop, both flips, rotation up to 10 degrees, translation up to 10 percent and mild brightness and contrast jitter. Evaluation uses a deterministic resize. Both use ImageNet normalization.
12. Take input size, batch size and gradient accumulation from the per-architecture profile so that the effective batch is always 32.
13. Optimize binary cross entropy over independent sigmoids, weighted per label by the ratio of negatives to positives and capped at 20.
14. Train stage one with a frozen backbone and the head only, at learning rate 3 × 10⁻⁴ for at most 10 epochs with patience 4.
15. Train stage two from that checkpoint with all parameters unfrozen, at the architecture-specific learning rate between 1 × 10⁻⁵ and 3 × 10⁻⁵, for at most 25 epochs with patience 6.
16. Both stages use AdamW with weight decay 10⁻⁴, one warm-up epoch followed by cosine annealing, mixed precision, and gradient-norm clipping at 1.0.
17. Select the epoch with the lowest weighted validation loss, stop early once the patience budget is exhausted, and restore that state.
18. Save both checkpoints together with architecture, label order and stage, and refuse stage two on any metadata mismatch.

## Algorithm A.7: Threshold Calibration, Pooled Out-of-Fold Evaluation and Model Comparison

**Input:**
20 fine-tuned checkpoints and their fold splits
**Output:**
Per-fold and pooled out-of-fold metrics, confusion matrices, comparison table and self-contained report

1. Reload each checkpoint and infer the test and validation split of its own fold without augmentation.
2. Convert the logits into five independent probabilities with a sigmoid and record the forward time per sample.
3. Calibrate every label on the validation split by maximizing F1 over the grid 0.05 to 0.95 in steps of 0.01, resolving ties towards 0.50.
4. Fall back to 0.50 for labels that carry only one target value in validation, and record a warning.
5. Score the test split twice: once with the fixed threshold 0.50 and once with the calibrated per-label thresholds.
6. Report per decision rule exact match, Hamming loss and accuracy, precision, recall and F1 in micro, macro, weighted and per-sample averaging, average precision and ROC-AUC.
7. Report per label support, prevalence, precision, recall, F1, specificity, negative predictive value, Cohen's kappa, average precision, ROC-AUC and the four confusion counts.
8. Store one binary confusion matrix per label plus their sum as the micro-pooled overall matrix.
9. Export the predictions in long format, one row per image and label with target, probability and both decisions.
10. Aggregate each architecture over its five folds as mean and standard deviation of every metric.
11. Concatenate the five test splits into the out-of-fold set and verify that every image-label pair occurs exactly once.
12. Rescore the pooled set with both decision rules, where each image keeps the calibrated thresholds of its own fold.

# APPENDIX B. ADDITIONAL NUMERICAL BASELINE RESULTS

## Table B.1: Numerical values underlying Figure 3.

Table B.1: Numerical values underlying the label-wise confusion matrices shown in Figure 3. Counts are based on the pooled out-of-fold predictions of Swin Transformer V2 Base using the calibrated decision thresholds.

| Label | TN | FP | FN | TP |
| --- | --- | --- | --- | --- |
| Major road | 6,936 | 765 | 636 | 1,663 |
| Power plant | 7,952 | 421 | 550 | 1,077 |
| Rail | 7,087 | 615 | 682 | 1,616 |
| Substation | 5,845 | 1,661 | 789 | 1,705 |
| Storage tank | 7,284 | 864 | 527 | 1,325 |
